# Barriers to integration of behavioral and social sciences in the general medicine curriculum and recommended strategies to overcome them: A systematic review

**Published:** 2016-07

**Authors:** ZAHRA TABATABAEI, SHAHRAM YAZDANI, RAMIN SADEGHI

**Affiliations:** 1 School of Medical Education, Shahid Beheshti University of Medical Sciences, Tehran, Iran;; 2 Nuclear Medicine Research Center, Mashhad University of Medical Sciences, Mashhad, Iran

**Keywords:** Integration, Social sciences, Behavioural sciences, Curriculum, Barriers

## Abstract

**Introduction:**

The integration of behavioral and social sciences (BSS) into the curriculum of medical students in order to equip them with the necessary knowledge, skills and attitudes is an essential issue, emphasized in many researches. Our aim is to investigate the barriers to integrate BSS into the general medicine curriculum as well as the recommended strategies to overcome such barriers through a systematic review of literature.

**Methods:**

PubMed, ERIC, Scopus, CINAHL, Google Scholar, and OPENGREY were searched for studies on the barriers to integration of BSS into the general medicine curriculum as well as the strategies employed to overcome them until August 28, 2015.

**Results:**

Sixteen relevant studies were included and the related domains were categorized as barriers and some strategies were recommended to overcome them. In addition, the quality of the included studies was assessed.

**Conclusion:**

Despite the prominent role of BSS in the effectiveness of health care, these sciences have not been included in the curriculum of medical students effectively. The identified barriers and the strategies used to overcome them should be considered for all integration programs. Future studies should focus on the process of BSS integration in the medical curricula and should evaluate the efficacy of this integration in more detail.

## Introduction

Healthcare systems throughout the world are constantly changing to meet the people’s emerging and diverse needs. In this regard, one of the domains that has been increasingly considered is the importance of behavioral and social sciences’ (BSS) contents and their relationship with clinical sciences in order to provide better care and effective treatment (1, 2).

Behavioral sciences deal with fundamental research studies on human behavior and aim to make use as much of available knowledge and skills as possible to improve our understanding of human behavior in order to enhance the quality of life. Social sciences are part of the humanities addressing different aspects of human’s social life. “The term behavioral and social sciences (BSS) incorporate the cognate disciplines, including psychology, sociology and anthropology” (3). A tangible example of BSS related to clinical sciences is the topic of physician-patient communication skills. In this situation, an effective therapeutic relation is established between the physician and the patient.  Awareness of social dimension of a patient's life plays a key role in the effectiveness of such a relationship.

Six domains of BSS needed to be integrated into the medical curriculum to enhance the medical students’ skills and competencies as suggested by Cuff & Vanselow (2004) are: mind–body interactions in health and disease, patient behavior, physician role and behavior, physician–patient interactions, social and cultural issues in health care and health policy and economics (1).

Although some scientific evidence suggests a close relationship between the behavioral and social factors and health, unfortunately, effective integration of these factors into the medical sciences and their correct interpretation, especially in the clinical performance of physicians, have not been accomplished. 

By an effective integration of BSS into the medical curriculum we mean the consistent inclusion of the related issues of these sciences to the basic and clinical courses in order to improve the physicians’ knowledge, skills and attitude which can subsequently have a great impact on their clinical practice. To do so, it is very important to identify the barriers for an effective integration and to carry out a proper plan to overcome them. The subject has been investigated by several studies.

Cuff & Vanselow (2004) studied the latest status of BSS content in the curricula of 126 medical schools in the U.S.A and 16 medical schools in Canada and identified the following items as barriers including inefficient leadership, managers’ resistance to change, lack of qualified experts among BSS faculty members, lack of adequate incentives in BSS and clinical faculty members and limited financial resources. In the remainder of their report, some strategies to overcome these barriers were proposed. Among the suggested strategies were career development award strategy, project awards which involve developing the curriculum in BSS and incorporating their contents in the medical licensing examination (1).

Russell, Teijlingen, Lambert & Stacy (2004) performed a study in the U.K. and identified that disagreement between the physicians and the BSS specialists and limitation of specialist manpower were the main barriers to implementing better education of BSS. In this study, some methods such as sufficient contents of BSS in the curriculum, teaching BSS by specialists and supervising the related courses in the medical curriculum by BSS specialists were proposed to overcome the barriers (4).

Another study was performed by Litva & Peters (2008) in the U.K. This work identified the following barriers for the integration of BSS into the general medical curriculum: reluctance to change the current curriculum, lack of qualified experts and faculty members to teach the related courses, limited time or space of the curriculum, traditional approach to medical assessment, reluctance of the predominant traditional model in medical education to change and the existence of a hidden curriculum. The study also refers to medical specialists’ misconception of the effective role of BSS in the medical curriculum and medical students’ increasingly negative attitudes among other factors. They finally noted that overcoming these barriers requires a deep commitment of clinical faculty members and BSS specialists (5-7).

The Report of the BSS Expert Panel published by AAMC (2011) identified the following items as barriers to integration: biomedical perspective, staffing issues, tight curriculum space, lack of standard curricula, content modules, clear learning objectives, training resources and some other obstacles. Moreover, some strategies to overcome these barriers including translating the content of BSS into the individual medical practice, trying to find out some ways to make BSS contents more integrated, relevant, and applicable and identifying successful educational strategies were proposed (6).

Hence, all of the conducted studies about BSS point to the increasing importance of integration of such measures into the general medical curriculum in different forms. Due to the existing role of BSS in the general medical curriculum and serious difficulties in accomplishing the task of integration, there need to be some systematic designs for integrating BSS into the curriculum of general medicine.

Therefore, the aim of this study is to conduct a systematic review of the available literature in order to identify current barriers to effective integration of BSS in the medical curriculum. The study also tries to examine proposed strategies to overcome the barriers. The results of this study provides managers and educational policy makers with better planning to achieve effective integration and to improve physicians’ knowledge, skills and attitudes in dealing with their patients which, in turn, results in prevention of diseases, more effective diagnosis and treatment. 

## Methods

Considering the three components of population, intervention and outcome, our research question was “What are the existing barriers regarding the integration of BSS into the general medical school curriculum and how can we overcome them?”

PubMed & Scopus, ERIC & CINAHL, Google Scholar, and OPENGREY (Grey literature) were searched to gather the relevant studies. 

A group consisting of three persons was formed to go through different stages of the systematic review. In our initial search, we combined the Medical Subject Headings (MeSH( term with variations of relevant text words (e.g., “behavioral”, “curricular” and “curricula”). The assignment of other MeSH terms was inconsistent. A thorough search was conducted in the intended databases using the following keywords: ("Behavioural science" [MeSH] or "behavioural science [text words]" or "social science" [MeSH]) and ("medical" [MeSH] or "medicine" [MeSH]) and ("curriculum" [MeSH] or "curricular" [text words] or "curricula" [text words]).

According to the search terms, our search, which included all related papers to BSS on medical curriculum and was wider than the intended outcome of study, was first conducted throughout all databases with a high-sensitivity. No language or time limit was exerted on the search. The last search was carried out on August 28, 2015.

The duplicated articles as well as the irrelevant titles or abstracts were removed. All titles and abstracts of the first search hits were evaluated independently by the first two authors and the articles that were in compliance with our research question were screened for full-text review. In case of any disagreement between the two authors, the third author’s opinion was considered.

After that, the studies that were in compliance with the inclusion and exclusion criteria were selected and included in our study. Table 1 shows the inclusion and exclusion criteria of our systematic review.

**Table1:** Inclusion and exclusion criteria

**Criteria**	**Inclusion**	**Exclusion**
**Population**	Medical students	Students of other health related disciplines
**Intervention**	General medicine curriculum including formal undergraduate training in the form of mandatory courses, elective courses, integrated themes, workshops, short exposures, longitudinal programs, integration BSS	Other medical discipline curricula, postgraduate training
**Outcome**	Identification of barriers and strategies to overcome them	Other outcomes including teaching methods, program contents, etc.
**Study type**	All quantitative and qualitative studies which evaluated outcomes, survey reports on prevalence of courses and related activities	Review articles and non-research reports

Finally, the second generation search in the relevant list of papers was performed by ancestry searching (reference lists of the included studies) and forward tracing (using the “cited by” tools of Scopus and Google Scholar). The corresponding authors of the studies were also contacted for any additional information or full-text request. 

**Data collection process**

Relevant information was extracted from the included studies: titles, authors, publication years, barriers and recommended strategies to overcome them. To minimize bias, the first two authors reviewed each included article independently. Studies passing the first screening were retrieved and evaluated by the first two authors independently. All conflicts were resolved by a discussion between the first two authors or, if necessary, by the third author’s intervention.

**Quality assessment and analysis**

In order to evaluate the quality of the included studies, the Medical Education Research Quality Index (MERSQI) tool was used. "The MERSQI, developed by Reed and colleagues (2007), has been shown to have content validity; inter-rater, intra-rater, and internal consistency reliability; criterion validity; and predictive validity. The MERQSI evaluates six domains of study quality: design, sampling, type of data, validity, data analysis, and outcomes. Another major advantage of the MERSQI is that it is easily applied to any medical education study, regardless of design, method, or outcome (8)".

The data were abstracted independently by the first two authors and input to qualitative tables for review. In this case, the data are the barriers to BSS integration into the general medicine curriculum and recommended strategies to overcome them. 

The identified barriers in each study were categorized according to relevant domains and each domain was categorized accordingly. The same strategy was also followed to categorize the proposed strategies to overcome barriers. We resolved differences in Quality assessment and analysis through consensus agreement.

The included studies were heterogeneous in several aspects and didn’t have any common quantitative outcome variables; consequently, we did not perform meta-analysis in the current study. 

## Results

**Study selection**

**Figure 1:**
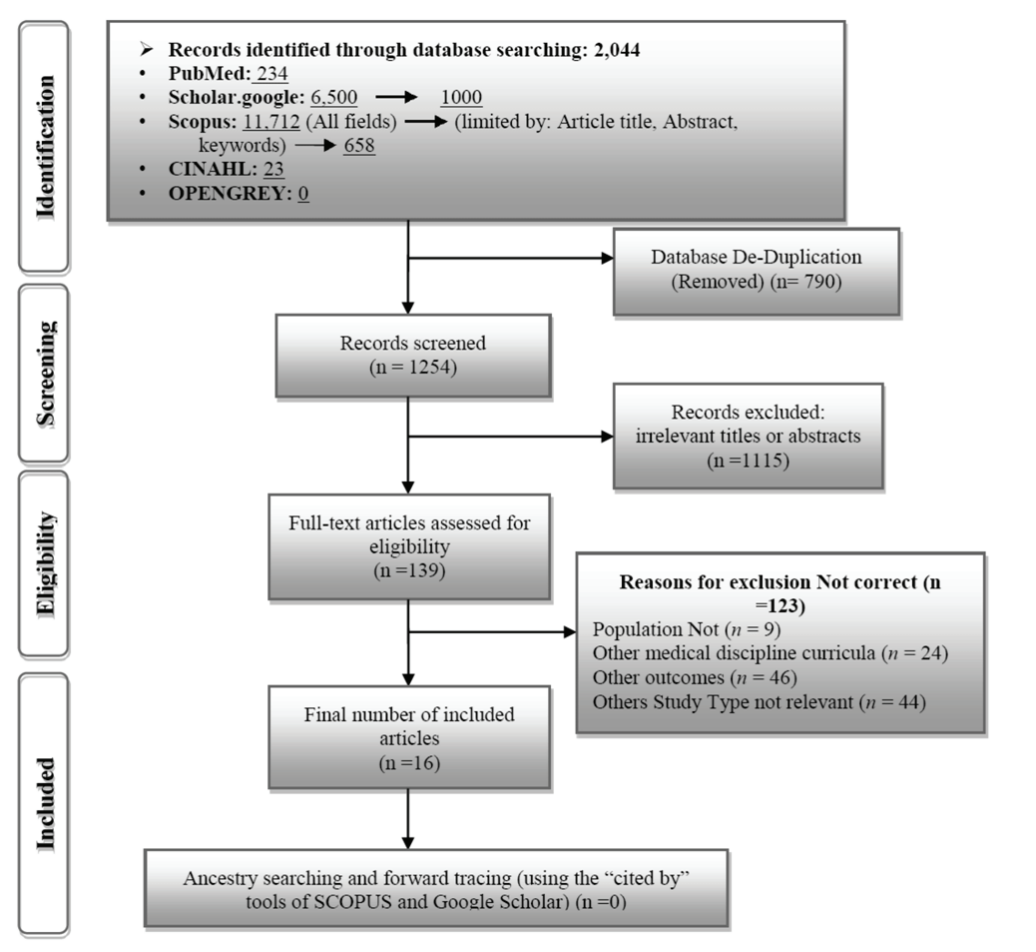
Search results of the systematic review until Aug. 2015

The search strategy of our systematic review is shown in PRISMA flow diagram (9) (Figure 1). The first search yielded 2,044 hits on integration of BSS into the medical curriculum in which there were 790 identical results and hence were removed from our initial list. In the next step, considering our research question, the titles and abstracts of the remaining results were checked and 1115 articles were removed due to irrelevant titles or abstracts. Hence, 139 articles were screened for full-text review. Finally, 16 relevant studies that were in compliance with the inclusion and exclusion criteria were selected and entered into the systematic review. It should be noted that ancestry searching and forward tracing did not yield any additional studies.

**Study** **Characteristics**

Table 2 shows the characteristics of the included studies.

**Table 2:** Characteristics of the16 included studies until Aug. 2015

**Study characteristics**	**No. of studies**
**Type of study **	
Single group cross-sectional	15
Single group pretest and posttest	1
**Type of sciences integrated**	
Behavioral sciences	4
Social sciences	1
Behavioral & social sciences	11
**Outcome**	
Barriers	16
Strategies to overcome barriers	12
**Country**	
United States	8
UK	5
Other	3

**Barriers and strategies to overcome them**

tables 3 and 4 show the qualitative data analysis of barriers and the recommended strategies to overcome them. "The data analysis phase includes ordering, coding, categorizing, thoroughly analyzing, impartially interpreting, and then summarizing the data found in the articles selected for inclusion (10, 11) ". An approach to data analysis that included a constant comparison method consisting of data reduction, data display, and data comparison, was used in this study (11) as:

1. Data reduction: In this stage, first, the semantic units related to barriers to integration and strategies to overcome them were extracted from primary sources and then ordered accordingly.
2. Data display: In this stage, the related codes to semantic units were extracted and categorized.
3. Data comparison: Finally, the extracted codes were analyzed, summarized and the related items were integrated and unified.

**Table 3:** Domains and components of barriers in the 16 included studies

**No.**	**Domains of ** **barriers**	**Components of barriers**
1	Inefficient leadership	1-1 Lack of the required knowledge and attitude about the importance of integration and consequently less motivation for the development of planning (1, 5) 1-2 Absence of the career development programs in the BSS (12) 1-3 Lack of support from social and behavioral scientists and clinicians for curriculum design and its development (1)
2	Problems related to BSS faculty members	2-1 Lack of the qualified expert faculty members and lack of relationship with external BSS investigators (1, 4, 5, 13, 14) 2-2 Logistic problems due to separate BSS faculty among multiple departments marginalizing of BSS faculties (1, 14, 15) 2-3 Lack of the necessary knowledge and experience in clinical medicine (16)
3	Problems related to clinical faculty members	3-1 Lack of adequate incentives due to insufficient support (1) 3-2 Lack of right knowledge about and proper attitude toward the importance and dynamics of BSS and their relationship with medical care (1,3,5,13,14,16,17) 3-3 Lack of well-trained and experienced faculty members for essential content training of BSS (4, 16) 3-4 Induction of negative attitudes in medical students towards BSS (4, 5)
4	Limited financial resources	4-1 to improve and develop a new content integration in the high-quality instructional programs (1, 18) 4-2 to perform more effective teaching techniques (1, 13, 16) 4-3 to assess students’ performance considering the effectiveness of teaching BSS (1, 18) 4-4 to teach both BSS and clinical faculty members and to support the development of new curriculum considering the time-consuming nature of the process (1, 13, 18)
5	Problems related to the curriculum	5-1 Existence of a hidden curriculum and lack of transferring BSS role-modeling during clinical courses (19, 20) 5-2 Lack of a standard model including appropriate contents with prioritized issues, effective teaching methods and appropriate evaluation systems (1, 4, 16, 21, 22) 5-3 Lack of a BSS database related to clinical practice (1) 5-4 Lack of a clear educational objectives for BSS and its relation with clinical practice (15, 22) 5-5 Lack of a systematic integration of BSS in all stages of the medical school curriculum (19, 22, 23) 5-6 Limited “time” or “space” of the curriculum considering the wide range of BSS (5, 12, 18, 22) 5-7 Reluctance of the predominant traditional model in medical education to change and its heterogeneity with the BSS mindset (5,12,13,19)
6	The conflict between BSS faculty members and clinicians	6-1 Discordant views between BSS and clinical faculty members and lack of commitment in these two groups to understand the relationship between clinical sciences and BSS (3, 4, 12)
7	Negative attitude of students	7-1 Lack of interest in the students due to failure to understand the relevance of BSS to clinical medicine (16, 18)

**Table 4:** Domains and components of strategies suggested to overcome barriers in the 16 included studies

**No.**	Domains of recommended strategies	Components of recommended strategies
**1**	Inefficient leadership	1-1 Using career development award strategies (1) 1-2 Awarding projects which involve in curriculum development (1) 1-3 Supporting BSS and clinical faculty members to design, develop and assess the curriculum at national and institutional levels (3, 13)
**2**	Problems related to BSS faculty members	2-1 Establishing departments of BSS within medical faculties (13, 15) 2-2 Forming larger core of BSS faculty members (13, 22) 2-3 Inviting adjunct BSS specialists to assess and develop the related courses in medical school curriculum (4)
**3**	Problems related to clinical faculty members	3-1 Training clinical faculty members for a deep understanding of the nature and the importance of BSS in clinical practice (4, 13) 3-2 Training clinical faculty members so that they can transfer these sciences to students through role-modeling, in such away so that the students accept these roles and values (12)
**4**	Limited financial resources	-
**5**	Problems related to the curriculum	5-1 Systematic and applied integration of a prioritized list of BSS into all steps of curriculum and continuous development of the curriculum (12, 16, 23) 5-2 Limiting the content to knowledge and skills that every medical student needs to know, regardless of final specialty and ensuring the sufficient content of BSS within the curriculum of all students (4, 12) 5-3 Establishing a BSS database which includes all available standards in order to be able to conform to the general medicine curriculum goals and achieve successful educational strategies (1, 4) 5-4 Integration of BSS thematic contents in the student assessment (1) 5-5 Substantially less time is needed to include the BSS due to their inherent nature (12, 13) 5-6 Developing a competitive environment using the new educational technologies in order to motivate the students (16, 19, 21)
**6**	The conflict between BSS faculty members and clinicians	6-1 Establishment of a mutual understanding and commitment between BSS and clinical faculty members to uncover the effects of different hidden curricula and to make a deeper penetration of BSS into clinical practice (5, 19) 6-2 Establishing an effective communication and close cooperation between BSS and clinical faculty members to reach a common language and understanding along with team work to achieve a more effective way of learning (3, 12, 13, 16)
**7**	Negative attitude of students	-

Table 5 shows included studies which had information in various domains.

**Table 5:** Distribution of barriers and strategies to overcome them in the 16 included studies

**Domains of ** **barriers and recommended strategies**	**Total articles studied the barriers**	**Total articles that recommend some strategies**
Inefficient leadership	3	3
Problems related to BSS faculty members	7	4
Problems related to clinical faculty members	8	3
Limited financial resources	3	-
Problems related to the curriculum	13	8
The conflict between BSS faculty members and clinicians	3	6
Negative attitude of students	2	-

**Quality assessment and** **analysis**

The quality of the included studies was assessed with the Medical Education Research Study Quality Instrument (MERSQI) (7) (Appendix 1).

**Appendix 1:** Quality assessment  of the included studies  utilizing the Medical Education Research Quality Index (MERSQI) tool, until Aug. 2015

**Study author** **and year**	**Country**	**Type of study**	**Type of sciences had been integration**	**Sampling**	**Type of data**	**Validity of evaluation instrument**	**Data analysis**	**Outcomes**	**Type of barriers**	**Strategies suggested**
**Cuff P.A. & Vanselow N.A., 2004**	USA	Single group cross-sectional	Behavioral & Social Science	>2^*^ <50 or not reported^**^	Assessment by study participant	Reported	Data analysis appropriate for study design and type of data^**^^*^ Beyond descriptive analysis^***^^*^	Opinions, general facts	1-1, 1-3, 2-1, 2-2, 3-1, 3-2, 4-1, 4-2, 4-3, 4-4, 5-2, 5-3	1-1, 1-2, 5-4, 5-3
**Russell A., Teijlingen E.V., et al, 2004**	UK	Single group cross-sectional	Behavioral & Social Science	>2^*^ 50-74^*^^*^	Assessment by study participant	Not reported	Data analysis appropriate for study design and type of data^**^^*^ descriptive analysis only ^***^^*^	Opinions, general facts	2-1, 3-3, 3-4, 5-2, 6-1	2-3, 3-1, 5-2, 5-3
**Litva A. & Peters S., 2008**	UK	Single group cross-sectional	Behavioral & Social Science	>2^*^ 50-74^*^^*^	Assessment by study participant	Not reported	Data analysis appropriate for study design and type of data^**^^*^ descriptive analysis only ^***^^*^	Opinions, general facts	1-1, 2-1, 3-2, 3-4, 5-6, 5-7	6-1
**Benbassat J., Baumal R., et al, 2003**	Israel & Canada & Rhode Island	Single group cross-sectional	Behavioral & Social Science	>2^*^ N/A^****^^*^	Assessment by study participant	Not reported	Data analysis appropriate for study design and type of data^**^^*^ Beyond descriptive analysis^***^^*^	Opinions, general facts	2-3, 3-3, 5-2, 7-1	5-1, 5-6, 6-2
**Peters S. & Livia A., 2006**	UK	Single group cross-sectional	Behavioral & Social Science	>2^*^ 50-74^*^^*^	Assessment by study participant	Not reported	Data analysis appropriate for study design and type of data^**^^*^ descriptive analysis only ^***^^*^	Opinions, general facts	3-2, 6-1	1-3, 6-2
**Hurster M., 1981**	USA	Single group cross-sectional	Behavioral Science	>2^*^ <50 or not reported^**^	Assessment by study participant	Not reported	Data analysis appropriate for study design and type of data^**^^*^ Beyond descriptive analysis^***^^*^	Opinions, general facts	4, 5-6, 7-1	-
**Griffiths J. A., 1978**	UK	Single group cross-sectional	Behavioral & Social Science	>2^*^ N/A^****^^*^	Objective measurement	N/A^****^^*^	Data analysis appropriate for study design and type of data^**^^*^ descriptive analysis only ^***^^*^	Opinions, general facts	2-2,5-4	2-1
**Satterfield J.M., Adler S.R., et al, 2010**	USA	Single group cross-sectional	Behavioral & Social Science	>2^*^ <50 or not reported^**^	Assessment by study participant	Reported	Data analysis appropriate for study design and type of data^**^^*^ Beyond descriptive analysis^***^^*^	Opinions, general facts	1-2, 5-6, 5-7, 6-1	3-2, 5-1, 5-2, 5-5, 6-2
**Post D.M., Stone L.C., et al, 2008**	USA	Single group cross-sectional	Behavioral Science	>2^*^ <50 or not reported^**^	Assessment by study participant	Not reported	Data analysis appropriate for study design and type of data^**^^*^ Beyond descriptive analysis^***^^*^	Opinions, general facts	5-5	5-1
**Satterfield J.M., Mitteness L.S., et al, 2004**	USA	Single group cross-sectional	Behavioral & Social Science	1^*^ N/A^****^^*^	Assessment by study participant	N/A^****^^*^	Data analysis appropriate for study design and type of data^**^^*^ descriptive analysis only ^***^^*^	Opinions, general facts	2-1, 3-2, 4-2, 4-4, 5-7	1-3, 2-1, 2-2, 3-1, 5-5, 6-2,
**Peterson C.D., Rdesinski R.E., et al, 2011**	USA	Single group cross-sectional	Behavioral & Social Science	>2^*^ ≥75^*^^*^	Assessment by study participant	Reported	Data analysis appropriate for study design and type of data^**^^*^ descriptive analysis only ^***^^*^	Opinions, general facts	5-1, 5-5, 5-7	5-6, 6-1
**Jacobs J.L., Lee M.T., et al, 2005**	USA	Single group cross-sectional	Behavioral & Social Science	>2^*^ ≥75^*^^*^	Assessment by study participant	Not reported	Data analysis appropriate for study design and type of data^**^^*^ descriptive analysis only ^***^^*^	Opinions, general facts	5-2	5-6
**Sheldrake P., 1973**	Edinburgh, UK	Single group pretest and posttest	Behavioral Science	>2^*^ <50 or not reported^**^	Assessment by study participant	Not reported	Data analysis appropriate for study design and type of data^**^^*^ descriptive analysis only ^***^^*^	Opinions, general facts	3-2	-
**Haidet P., Kelly A. & Chou C., 2005**	USA	Single group cross-sectional	Behavioral Science	>2^*^ <50 or not reported^**^	Assessment by study participant	Reported	Data analysis appropriate for study design and type of data^**^^*^ Beyond descriptive analysis^***^^*^	Opinions, general facts	5-1	-
**Cohen R. &Kelner M., 1976**	Canada	Single group cross-sectional	Behavioral & Social Science	>2^*^ 50-74^*^^*^	Assessment by study participant	Not reported	Data analysis appropriate for study design and type of data^**^^*^ Beyond descriptive analysis^***^^*^	Opinions, general facts	5-2, 5-4, 5-5, 5-6	2-2
**Obot I.S., 1988**	Nigeria	Single group cross-sectional	Social Science	>2^*^ 50-74^*^^*^	Assessment by study participant	Not reported	Data analysis appropriate for study design and type of data^**^^*^ descriptive analysis only ^***^^*^	Opinions, general facts	2-1, 2-2, 3-2	-

^*^No. of institutions studied

^**^ Response rate, %

^***^ Appropriateness of analysis

^****^ Complexity of analysis

^*****^ Not applicable

## Discussion

In the current study, the available literature was searched for the integration of BSS into the medical curriculum and strategies to overcome them and the results were presented in a systematic review format.

As shown in tables 3 and 4 several domains could be identified in the literature regarding the barriers of BSS integration in medical curricula and the strategies to overcome them:

***Ineffective leadership***

One of the main barriers to integrate BSS into medical curriculum is inefficient leadership. Planning to improve infrastructures and improving the quality of curriculum primarily depends on program managers’ attitude. Therefore, the most important component of this barrier seems to be lack of proper knowledge and attitude towards the importance of BSS. This can reduce the incentive and build up resistance to the process of BSS integration into medical curriculum. A good example in this regard is refusing to allocate enough education time for BSS under the pretext of limited time and space of curriculum (1, 5, 24).

Our study revealed that managers’ and educational policy makers’ lack of knowledge and positive attitude prevent them from supporting the professional development of BSS. That is exactly why they are mostly reluctant to develop faculties and departments related to these sciences in the body of medical schools. To improve the process of continuous development and assessment of the curriculum and to further integrate BSS into clinical practice, cooperation between BSS faculty members and clinicians seems to be essential (1, 5, 13). The motivation to cooperate is achievable through improvement of knowledge, skills and attitudes concerning the importance of BSS. Applying incentive and supportive policies such as special rewards for effective projects of BSS integration into the medical curriculum can also be very fruitful (1). According to the standards in medical education, in order to have more effective programs and policies for the integration of BSS into the medical curriculum, the integration process needs be consistent with the advancement of medical sciences, the changing demographic characteristics and the community health. This would require systematic programs from managers and educational policy makers (25).

***Limited financial resources***

Another barrier is limited financial resources. All the required education and training for integration process need enough budget allocation. It is clear that systematic planning to reform the predominant traditional programs is needed. To do this, all steps from providing the necessary infrastructures to implementation of program needs sufficient allocation of financial resources. Some other items that need financial resources are training clinicians to understand the nature and the importance of BSS in clinical practice and also training BSS faculty members to understand the necessity of getting knowledge and changing attitude about clinical medicine. The time-consuming process of integration and requirement for a continuous and costly cooperation between clinical and BSS faculty members underscore the importance of enough budgeting. In addition, effective teaching methods, providing facilities and the required training resources, content determination and continuous assessment of student performance and evaluation of the instructional program- in order to check the effectiveness of integration- are costly, too (1, 13, 18). Enough financial support is not achievable unless the integration is moved from the margin into the mainstream of medical education. Therefore, allocating financial resources to have a comprehensive support is expected to be considered seriously by educational managers.

***Problems related to clinical faculty members***

Other barriers are problems related to clinical faculty members. It should be noted that since BSS are outside the knowledge scope of clinical faculty members, increasing their motivation, developing their skills and changing their attitude need to be supported by program managers (1, 24, 26). Lack of knowledge and skill of clinical faculty members regarding BSS would lead to misunderstanding in medical students. This can decrease their motivation, strengthen their negative attitudes, increase apathy and finally make them unable to understand the role of these sciences properly (4, 5, 16, 18). Hence, students cannot perceive the patients’ problems as part of a widespread social and environmental framework.

Another major weakness of the clinical faculty members’ attitudes toward BSS is the incompatibility of the dominant biomedical culture with the values of BSS. From a clinical perspective, human diseases are related to a mismatch between anatomical and biomedical issues. Considering the dynamics of social and behavioral components, the role of these factors is neglected by clinical faculty members and they ignore the inseparable relation of BSS with medical care (1, 3-5,12-14,16,17). Overcoming the above-mentioned barriers is possible only by improving the knowledge, skills and attitude of clinical faculty members and consequently the acceptance of these role models by their students. Medical students are advised to see beyond the traditional cause-effect relationship of the diseases and they are expected to be aware of issues beyond the biomedical approaches (i.e. social dimension of the human pathologies). This would definitely improve the outcome of their future medical practice (4, 12, 13).

***Problems related to BSS faculty members***

Not only is the number of BSS faculty members limited, the existing members usually remain on the sidelines and are usually scattered in different departments which make them inefficient (1, 4, 5, 13-15). In order to overcome this barrier, professional development of BSS faculty members and establishment of specialized departments for the BSS members within medical schools seems to be mandatory. This can only be accomplished by qualified managers and educational policy makers (13, 15, 22).

Another important problem is BSS members’ lack of necessary knowledge and experience in clinical medicine (16, 27). Improving the knowledge of BSS faculty members on the clinical sciences and improving their attitude toward them are of utmost importance for better employment of knowledge, experience and skills of BSS faculty members. This is only achievable by closer cooperation between BSS and clinical faculty members (4, 26).

This can lead to a sharing their resources and contents as well as developing a common language and understanding which can finally lead to discovering the hidden curriculum (20, 26).

***Problems related to the curriculum***

Some barriers associated with implementing methods for integrating these sciences effectively include reluctance of both biomedical model and the predominant traditional methods and their discordance with the BSS mindset (5,12,13,19,28,29). To show the superiority of integrated curriculum over the current one, it is necessary to identify the existing curriculum problems and adopt the appropriate strategies to solve them.

One of these curriculum-related problems is unclear educational objectives for BSS and their relation with clinical practice (15, 22). Other problems include lack of a standard model for the curriculum, lack of effective teaching methods and efficient evaluation systems. In order to overcome the curriculum-related problems clarifying the objectives is of primary importance and designing a standard curriculum is of secondary importance. To design a standard curriculum, it is necessary to create a database which includes all aspects of an effective integration from a prioritized content to determine the type of evaluation systems (1, 2, 4, 16, 23). All of the contained information in this database, according to the world standards for medical education, is advised to comply with the advancement of medical sciences, changes in the demographic characteristics and the national and regional health needs (1, 25). In this way, everyone can determine his/her goals according to successful strategies clearly and comply with standards (4).

Currently, the necessary question of the inclusion of BSS in a curriculum has been replaced with how to include contents of BSS in a more focused, more relevant, more applicable and systematic way in all stages of the medical school curriculum (6, 30, 31). Despite the achievements in this field, there is still a considerable lack of coordination and agreement on the kinds of contents, effective teaching methods and assessment of the students' performance (1, 4, 16). Considering the wide range of BSS and the limitation of time and space in the curriculum, role-modeling is the best strategy to convey BSS messages in the covers of clinical courses (5, 12, 13, 18, 22). Furthermore, in order to propose more practical solutions to overcome this problem, one can integrate these sciences to the curriculum by the means of thread model. In this model, intended concepts are integrated as meta-competency. To do this, learning objectives which are almost cognitive and affective are achieved with a little content coverage throughout the curriculum.

Given all of the above, we are required to limit the content to essential knowledge and skills regardless of their specific expertise, and make sure that all students have access to this content (4, 12). It is essential to overcome the barriers associated with lack of effective teaching methods, to employ new educational technologies and develop a competitive environment in the curriculum to increase the students’ motivation (16, 21, 23). Finally, in order to develop these sciences systematically and to use the above-mentioned successful strategies, educational managers are required to improve and develop the goals of the program and take into account the existing standards.

## Conclusion

Considering the rapid change of social indices and progress of medical sciences, the importance of BSS will have to be more emphasized because of the changes in demographic characteristics and epidemiological issues. Such changes should comply with health needs. This, in turn, necessitates standardization and effective integration of the medical education programs.

In this regard, effective integration of these sciences into the medical curriculum needs more systematic and coordinated actions by program managers. If strongly supported and carefully planned, it leads to an increase in the spirit of cooperation and the development of a common understanding between clinicians and BSS faculty members. 

Such an effective relation results in prioritizing the related content areas of BSS and integrating them in terms of training topics. Systematic integration of BSS increases the perception and attitude of clinical faculty members as well as medical students. This improves the physician-patient relation and finally results in a more effective health care. Thus, faculty members and experts in the field of BSS are more needed and this need can be met through establishing specialized BSS departments within the medical schools.

Therefore, the first step toward an effective integration of these sciences into the medical curriculum is identification of the current barriers along with the proposed strategies to overcome them.

Future studies should focus on the process of BSS integration into the medical curricula and should evaluate the efficacy of this integration in more details. For instance, it should be tried to find out the effectiveness of such systematic integration into the outcomes of effective communication of physicians with their patients, and colleagues. Effective management of patients, effective treatment relationships and stress management in physician’s and patient’s lives are also of prime importance.

**Strengths and weaknesses**

Effective integration of behavioral and social sciences in the curriculum of medical students is of utmost importance and the effect of this integration on the clinical practice of medical students is evident. Introduction to barriers of integration and strategies to overcome them, which is the goal of our study, could promote the curriculum and have a dramatic effect on clinical interventions and patients’ treatment. Unavailability and oldness of some sources as well as limitations on some papers due to retrieval bias can be cited as ours weakness. 
